# The expression of the ubiquitin ligase subunit Cks1 in human breast cancer

**DOI:** 10.1186/bcr1278

**Published:** 2005-07-19

**Authors:** Merav Slotky, Ma'anit Shapira, Ofer Ben-Izhak, Shai Linn, Boris Futerman, Medy Tsalic, Dan D Hershko

**Affiliations:** 1 Department of Surgery A, Rambam Medical Center and the Technion–Israel Institute of Technology, Haifa, Israel; 2 Department of Pathology, Rambam Medical Center and the Technion–Israel Institute of Technology, Haifa, Israel; 3 Department of Medical Oncology, Rambam Medical Center and the Technion–Israel Institute of Technology, Haifa, Israel; 4 Unit of Clinical Epidemiology, Rambam Medical Center and the Technion–Israel Institute of Technology, Haifa, Israel; 5 Breast Health Institute, Rambam Medical Center and the Technion–Israel Institute of Technology, Haifa, Israel

## Abstract

**Introduction:**

Loss of the cell-cycle inhibitory protein p27^Kip1 ^is associated with a poor prognosis in breast cancer. The decrease in the levels of this protein is the result of increased proteasome-dependent degradation, mediated and rate-limited by its specific ubiquitin ligase subunits S-phase kinase protein 2 (Skp2) and cyclin-dependent kinase subunit 1 (Cks1). Skp2 was recently found to be overexpressed in breast cancers, but the role of Cks1 in these cancers is unknown. The present study was undertaken to examine the role of Cks1 expression in breast cancer and its relation to p27^Kip1 ^and Skp2 expression and to tumor aggressiveness.

**Methods:**

The expressions of Cks1, Skp2, and p27^Kip1 ^were examined immunohistochemically on formalin-fixed, paraffin-wax-embedded tissue sections from 50 patients with breast cancer and by immunoblot analysis on breast cancer cell lines. The relation between Cks1 levels and patients' clinical and histological parameters were examined by Cox regression and the Kaplan–Meier method.

**Results:**

The expression of Cks1 was strongly associated with Skp2 expression (*r *= 0.477; *P = *0.001) and inversely with p27^Kip1 ^(*r *= -0.726; *P *< 0.0001). Overexpression of Cks1 was associated with loss of tumor differentiation, young age, lack of expression of estrogen receptors and of progesterone receptors, and decreased disease-free (*P = *0.0007) and overall (*P = *0.041) survival. In addition, Cks1 and Skp2 expression were increased by estradiol in estrogen-dependent cell lines but were down-regulated by tamoxifen.

**Conclusion:**

These results suggest that Cks1 is involved in p27^Kip1 ^down-regulation and may have an important role in the development of aggressive tumor behavior in breast cancer.

## Introduction

The prognosis and clinical management of patients with breast cancer are commonly determined by traditional clinicopathological factors such as tumor size and grade, lymph node status, and the expression of receptors to estrogen (ERs) and to progesterone (PRs) and of Her2/neu [1]. Nevertheless, patients may have significantly different clinical outcomes despite similar clinicopathological features. Some of these differences may be attributed to alterations in the normal regulation of the cell cycle that ultimately lead to aggressive tumor behavior.

Among the various cell-cycle proteins, deregulation of p27^Kip1 ^expression was found to have a particularly important role in cancer [2]. Numerous studies have shown that down regulation of p27^Kip1^, an inhibitor of cyclin-dependent kinases, is associated with poor prognosis in many cancers, including breast, colorectal, prostate, and lung carcinomas [3-8]. The decrease in p27^Kip1 ^levels in these cancers was found to result from its rapid degradation by the ubiquitin–proteasome pathway, rather than from decreased protein synthesis or gene mutation [6,9,10].

The main rate-limiting regulator for p27^Kip1 ^degradation was identified as an SCF-type ubiquitin ligase complex that contains S-phase kinase protein 2 (Skp2) as the specific substrate-recognition subunit [11-13]. Skp2 specifically binds p27^Kip1 ^and targets it for degradation by the ubiquitin proteolytic system. The important role of Skp2 in controlling p27^Kip1 ^levels in some human cancers, including breast, prostate, colorectal, and oral squamous cell carcinomas, was recently emphasized [14-17]. Thus, increased expression of Skp2 in these tumors was associated with low p27^Kip1 ^levels, aggressive tumor behavior, and poor overall survival.

More recently, the essential role of cyclin kinase subunit 1 (Cks1) in facilitating the ubiquitin-mediated proteolysis of p27^Kip1 ^through interaction with Skp2 was discovered [18,19]. Cks1 is a member of the highly conserved family of Cks/Suc1 (*Schizosaccharomyces pombe *cell-cycle regulatory protein) proteins, which interact with Cdks (cyclin-dependent kinase), but its exact mechanism of action remained poorly understood until recently. Cks1 was found to be an essential cofactor for efficient Skp2-dependent ubiquitination of p27^Kip1^. The critical role of Cks1 in targeting p27^Kip1 ^for efficient degradation by Skp2 was emphasized by demonstrating the lack of p27^Kip1 ^ubiquitination and breakdown in the absence of Cks1 *in vitro *and the slow proliferation and accumulation of p27^Kip1 ^in Cks1 nullizyous mice *in vivo *[18,19]. Recent studies from our and other laboratories have investigated the expression of Cks1 levels in different human cancers and its relation to Skp2 and p27^Kip1 ^expression [20-24]. For example, we have shown that in colorectal cancer the expression of Cks1 protein levels correlated strongly with the expression of Skp2 protein levels and inversely with those of p27^Kip1 ^[23]. Furthermore, Cks1 expression was increased in poorly differentiated tumors and was strongly and independently associated with poor overall survival [24]. The role of Cks1 in breast cancer, however, is unknown.

In the present study, we investigated the expression of Cks1 in relation to Skp2 and p27^Kip1 ^and prognosis in breast cancer. We show that Cks1 expression is strongly associated with Skp2 expression and inversely associated with p27^Kip1 ^expression. Furthermore, our results suggest that Cks1 expression may be used as an independent prognostic marker for disease-free and overall survival in breast cancer.

## Materials and methods

### Patients and tissue samples

Tissue samples from 50 patients with primary breast carcinomas that had been operated on more than 6 years before the beginning of the study were collected for immunohistochemical studies, after we had obtained the approval of the institution's Human Investigation Committee. Patients presenting with metastatic disease were excluded from this study. Clinical and histological data were available for all of these patients. Long-term follow-up data were provided by patients' medical records and the Israel Cancer Registry.

### Cell cultures

Human breast cancer cell lines (MCF-7, T47D, and MDA-MB-231) were generously provided by Dr H Degani (Weizmann Institute of Science, Rehovot, Israel). Because Skp2 levels change during the cell cycle (being normally highest in the S phase and lowest in the G1 phase) we cultured the cells in different media under conditions of similar proliferation rate in all cell lines. MDA-MB-231 cells were grown in RPMI medium (Biological Industries, Beth Ha'emek, Israel) supplemented with 10% fetal calf serum, 100 Units penicillin, and 100 μg streptomycin per ml and 1 mM sodium pyruvate. MCF-7 breast carcinoma cells were cultured in Dulbecco's modified Eagle's medium (DMEM; Biological Industries) supplemented with 10% fetal bovine serum, 4.5 g/l glucose, antibiotics as described above, and 4 mM glutamine. The T47D cell line was cultured in RPMI medium supplemented with 10% fetal calf serum, 100 Units/ml penicillin, 100 μg/ml streptomycin, and 10 μg/ml insulin. All cell lines were cultured at 37°C in 5% CO_2_. Under these conditions, the proliferation rate of all cell lines was similar (21 to 22 hours doubling time).

### Immunoblotting

Samples containing 30 μg of protein were resolved by electrophoresis on a 12.5% SDS-polyacrylamide gel and were transferred to nitrocellulose membranes. The membranes were probed with affinity-purified rabbit polyclonal antibody directed against a 12-amino-acid synthetic peptide from the extreme C terminus of human Cks1 (gift of Dr A Hershko, Technion, Haifa, Israel). This antibody does not cross-react with Cks2. The antibody was diluted 1:200 in TBST (Tris-buffered saline and 0.1% Tween 20) containing 10% (w/v) nonfat dry milk. Membranes were also probed against the mouse monoclonal antibody directed against Skp2 (Zymed Laboratories Inc, South San Francisco, CA, USA) diluted 1:500, or with a rabbit polyclonal antibody directed against p27^Kip1 ^(Zymed) diluted at 1:250. Membranes were also probed with a mouse monoclonal antibody directed against Skp1 (Transduction Laboratories, Lexington, KY, USA; 1:1000 dilution). Since levels of Skp1 do not change in the cell cycle, this protein served as an internal control to normalize for loading of cellular protein. After being washed with TBST, the immunoreactive proteins were visualized with horseradish-conjugated IgG (Pierce, Rockford, IL, USA) diluted 1:10,000 and an enhanced chemiluminescence system (SuperSignal West Pico; Pierce). All immunoblot analyses were done at least twice.

### Immunohistochemistry

Immunohistochemical studies were performed on formalin-fixed tissue sections embedded in paraffin wax. Five-micron sections were deparaffinized with xylene and rehydrated in a series of ethanols.

For epitope retrieval, slides were heated in 1 mM ethylenediaminetetraacetic acid buffer (pH 8), either in a microwave oven at 92°C for 20 min (p27^Kip1^) or in an Antigen Retrieval Processor (Milestone Inc, Sorsiole, Italy) at 120°C for 8 min (Skp2 and Cks1). After they had cooled, the slides were washed in distilled water.

Skp2 staining was carried out in the NexES IHC Immunostainer (Ventana Medical Systems, Tucson, AZ, USA), in accordance with the manufacturer's instructions, using a monoclonal antibody (Zymed) diluted 1:100.

Slides for p27^Kip1^and Cks1 staining were treated for 10 min with 3% H_2_O_2 _in methanol to block endogenous peroxidase and for 30 min with 10% nonimmune rabbit serum to block nonspecific protein binding. The slides were then washed in water and soaked in washing buffer (pH 7.4, Optimax; Biogenex, San Ramon, CA, USA) for 5 min. For p27^Kip1 ^staining, slides were incubated overnight at 4°C with the monoclonal p27^Kip1 ^antibody diluted 1:500. Staining was completed with a Histostain-plus kit (Zymed) in accordance with the manufacturer's instructions. For Cks1 staining, slides were incubated with an affinity-purified rabbit polyclonal antibody directed against a 12-amino-acid synthetic peptide from the extreme C terminus of human Cks1 (described above) diluted 1:40 for 2 hours at room temperature. Staining was completed using a Super-sensitive Multilink Immunodetection kit (Biogenex) in accordance with the manufacturer's instructions. The color reaction product was developed with aminoethyl carbazole (AEC). All sections were counterstained with hematoxylin.

For negative controls, slide sections that were positive for staining were treated with 10% nonimmune rabbit serum (Zymed) instead of the primary antibody. No staining was observed in any of these controls.

The immunohistochemical slides were scored according to the percentage of tumor cells exhibiting nuclear staining. To define high and low protein expression, we used a cutoff of 50% p27^Kip1 ^and 10% for Skp2, which is the cutoff used in most previous studies [14,15]. The cutoff for Cks1 was 10%, similar to the level used for Skp2, as in previous studies. When stained, cells displayed similar intensities of nuclear staining regardless of the percentage of cells stained, and therefore the intensity of staining was not included in the score. The specificity of immunohistochemistry staining procedures for Skp2, Cks1, and p27^Kip1 ^was previously verified by comparing the protein levels as determined by immunohistochemistry with the protein levels determined by immunoblot analysis from the same tumor specimens [15,23].

### Statistical analysis

Statistical data analyses were performed using SPSS 11.0 statistical software package (SPSS Inc, Chicago, IL, USA). First, the relations between Skp2, Cks1, and p27^Kip1 ^and those between protein levels and various clinical and pathological features were studied using cross tabulation and Pearson's χ^2^. Survival curves were constructed using the Kaplan–Meier method and multivariate analysis by Cox regression; *P *values less than 0.05 were considered significant.

## Results

### Cks1 protein expression is directly related to Skp2 expression and inversely related to p27^Kip1 ^expression in human breast cancer

**Figure 1:**
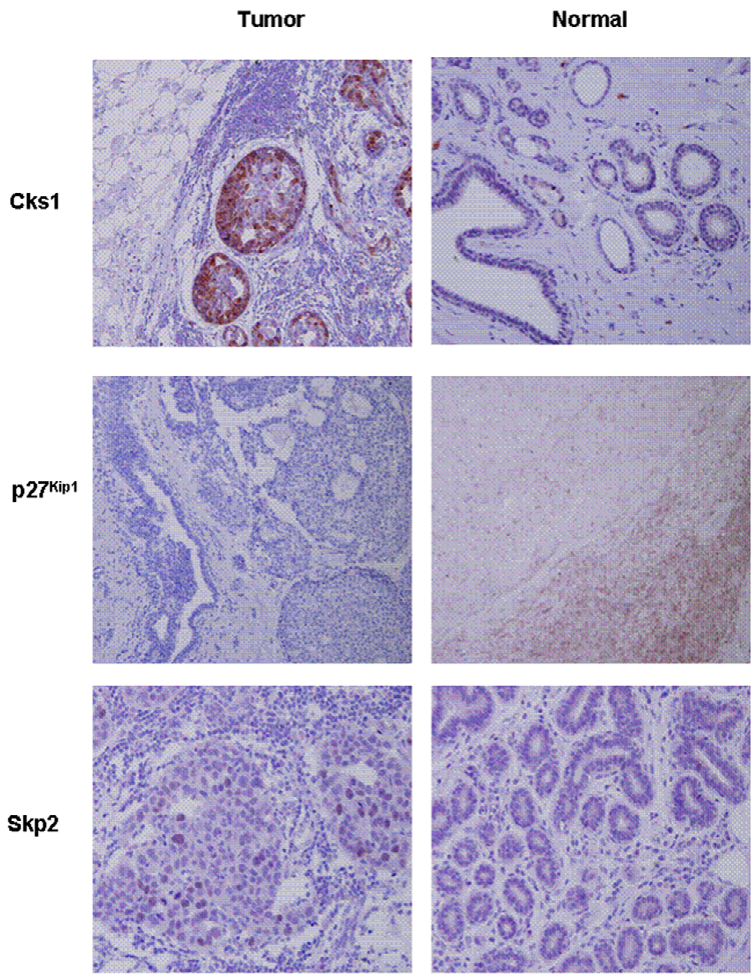
Representative immunohistochemistry slides for Cks1, Skp2, and p27^Kip1 ^staining in breast cancer. Tumor cells of a grade II invasive duct cancer exhibiting strong and diffuse nuclear staining for Skp2 (S-phase kinase protein 2) and Cks1 (cyclin kinase subunit 1) (× 300) and low p27^Kip1 ^staining. Note the inversely low Skp2 and Cks1 staining with high p27^Kip1 ^staining of normal adjacent breast tissue (× 400).

We studied the expression of Cks1, Skp2, and p27^Kip1 ^by immunohistochemistry in 50 tumor samples obtained from patients with breast cancer. The quality of immunostaining was good, with minimal background reactivity. Cks1 levels were very low or absent in all normal breast tissues adjacent to tumors. However, Cks1 levels were not uniformly high in all tumor specimens, being very high in some but virtually absent in others (Table 1). Given the important biochemical link between Cks1, Skp2, and p27^Kip1^, we examined the correlation between these proteins. Regression analysis of all 50 cases showed an inverse correlation between Skp2 and p27^Kip1 ^levels (*r *= -0.395; *P = *0.005). Cks1 expression was strongly related to Skp2 expression (*r *= 0.477; *P = *0.001) and inversely related to p27^Kip1 ^levels (*r *= -0.726; *P *< 0.001). Thus, strong relations were found between the expressions of all three proteins in 74% of the patients. High levels of Cks1 and Skp2 with low levels of p27^Kip1 ^were observed in 15 patients, and low levels of Cks1 and Skp2 with high levels of p27^Kip1 ^were observed in 22 patients. A typical representative immunohistochemical sample is shown in Fig. 1. In this tumor sample, cancer cells show strong nuclear staining for Cks1 and Skp2 and weak staining for p27^Kip1^, but high p27^Kip1 ^staining and low Cks1 and Skp2 staining in the normal surrounding breast tissue. In 13 of the 50 cases, the above relation was not found. In eight cases an inverse relation was found between Cks1 and p27^Kip1 ^expression but not between Skp2 and p27^Kip1 ^expression; in three cases, levels of both p27^Kip1 ^and Skp2 were high whereas Cks1 expression was low; and in another five cases, p27^Kip1 ^levels were low despite high Cks1 levels and low Skp2 levels. In another five cases, the differences between the three proteins were not significant.

### Cks1 overexpression is associated with poor tumor differentiation, young age, and negative ER/PR status

To examine the relation between Cks1 expression and common parameters associated with aggressive tumor behavior, we compared Cks1 levels with various clinicopathological features such as age, tumor size, pathological grade, lymph node status, and ER and PR expression (Table 1). A significant direct correlation was found between high Cks1 levels and loss of tumor differentiation (*r *= -0.421; *P = *0.012), negative ER expression (*r *= -0.356; *P = *0.013), and negative PR expression (*r *= -0.404; *P = *0.006). We also found a strong relation between Cks1 overexpression and patient age less than than 50 years (*r *= 0.327; *P = *0.049), but did not observe a significant correlation between Cks1 levels and lymph node status (*r *= 0.029; *P = *0.508) or tumor size (*r *= 0.192; *P = *0.333).

Examination of the association between Skp2 or p27^Kip1 ^and patients' clinicopathological characteristics showed a strong association between Skp2 expression and loss of tumor differentiation (*r *= -0.400; *P = *0.005), young age (*r *= -0.337; *P = *0.022), and negative ER or negative PR status (*r *= -0.589; *P = *0.001 and *r *= -0.674; *P = *0.001), respectively. Loss of p27^Kip1 ^was associated with loss of tumor differentiation (*r *= -0.323; *P = *0.032) and, respectively, loss of ER and PR (*r *= 0.298, *P = *0.048; *r *= -0.316, *P = *0.030 (Table 1)).

**Figure 2:**
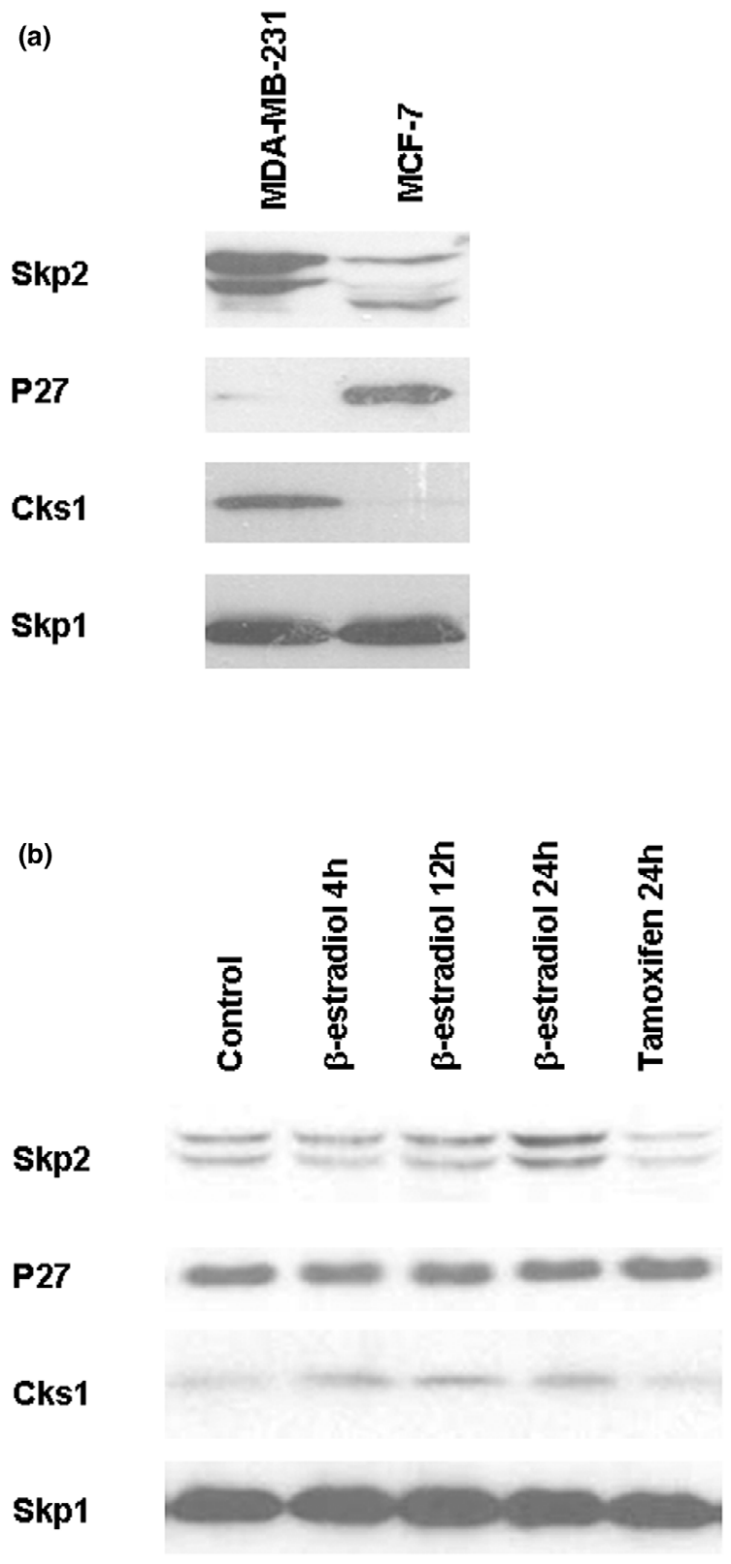
Immunoblots showing Cks1, Skp2, and p27^Kip1 ^proteins in breast cancer cell lines. (a) Levels of the three proteins Cks1 (cyclin kinase subunit 1), Skp2 (S-phase kinase protein 2), and p27^Kip1 ^were measured in MDA-MB-231 (an estrogen-independent cell line) and MCF-7 (an estrogen-dependent cell line). Skp1 levels were determined to confirm equal protein loading. (b)Effect of estrogen modulation on Cks1, Skp2, and p27^Kip1 ^expression in T47D cells (an estrogen-dependent breast cancer cell line) treated with estradiol (0.5 μM) or tamoxifen (10 μM). P27, p27^Kip1^.

Because of the strong association between Cks1, Skp2, and p27^Kip1 ^levels and the ER status of the tumors, we next examined the expression of these proteins in estrogen-dependent (MCF-7 and T47D) and estrogen-independent (MDA-MB-231) breast carcinoma cell lines, and the possible effects of estrogen modulation on the regulation of Skp2 and Cks1 expression. Basal levels of Cks1 and Skp2 were higher in estrogen-independent cells than in estrogen-dependent cells, whereas p27^Kip1 ^levels were lower (Fig. 2a). Interestingly, between the two estrogen-dependent cell lines, levels of Cks1 and Skp2 were higher in T47D cells, which phenotypicallly have poorer cellular differentiation (Fig. 2b). Treatment of T47D cells with estradiol increased Cks1 and Skp2 levels, whereas treatment with tamoxifen down regulated Cks1 and Skp2, resulting in an upregulation of p27^Kip1 ^levels (Fig. 2b).

### Cks1 overexpression is associated with poor disease-free and overall survival in human breast cancer

**Figure 3:**
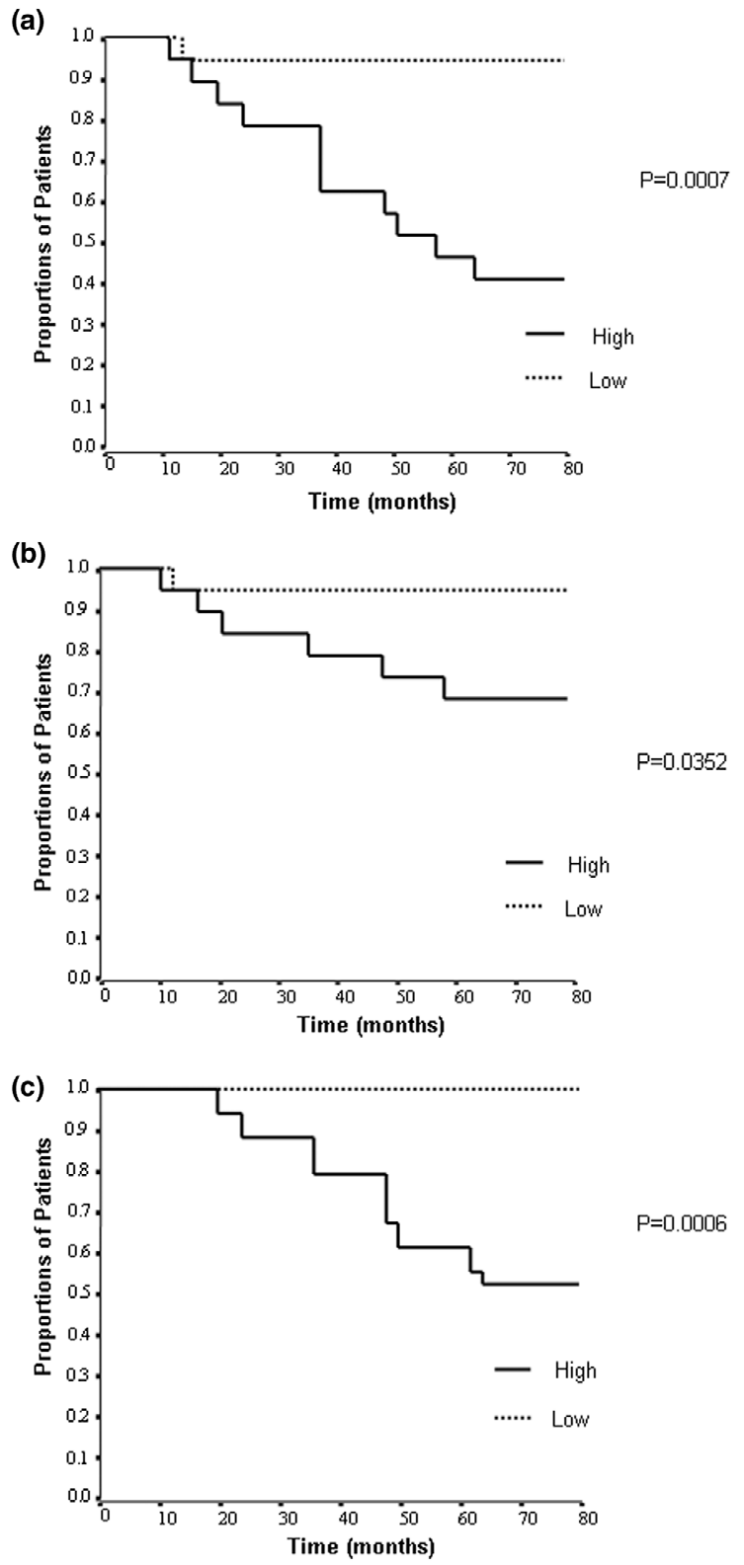
The association between Cks1 expression and disease-free survival. The expression of Cks1 (cyclin kinase subunit 1) was plotted in relation to overall (a), local (b), or distant (c) recurrence rates. Analyses were constructed using the Kaplan–Meier method. Protein expression was considered low if fewer than 10% of cell nuclei were stained and high if more than 10% of nuclei were stained.

To determine the association between Cks1 expression and prognosis, we plotted Kaplan–Meier curves for disease-free survival and overall survival. Complete 80-month follow-up data were available for all the patients. Patients presenting with high tumor Cks1 levels had significantly shorter disease-free survival rates than patients with low Cks1 levels (54 months vs 76 months, respectively; *P = *0.0007; Fig. 3a). There was only one case of disease recurrence in patients with low Cks1 levels, whereas in patients with high Cks1 levels, disease recurrence was observed in 11 (42% of patients). Similarly, Skp2 and p27^Kip1 ^were also good predictors for disease-free survival (*P = *0.0014 and *P = *0.0191, respectively; data not shown).

We also examined the prognostic role of Cks1 in predicting local or distant relapse disease-free survival (Fig. 3b,c, respectively). Patients with high Cks1 levels had both higher local recurrence rates (*P = *0.0352) and more distant relapse rates (*P = *0.0006) than patients with low Cks1 levels. Skp2 was also found to be a good predictor for local (*P = *0.0051) and distal (*P = *0.003) relapse rates, while p27^Kip1 ^expression was a predictor for distant relapse (*P = *0.031) but not for local recurrence (*P = *0.136).

**Figure 4:**
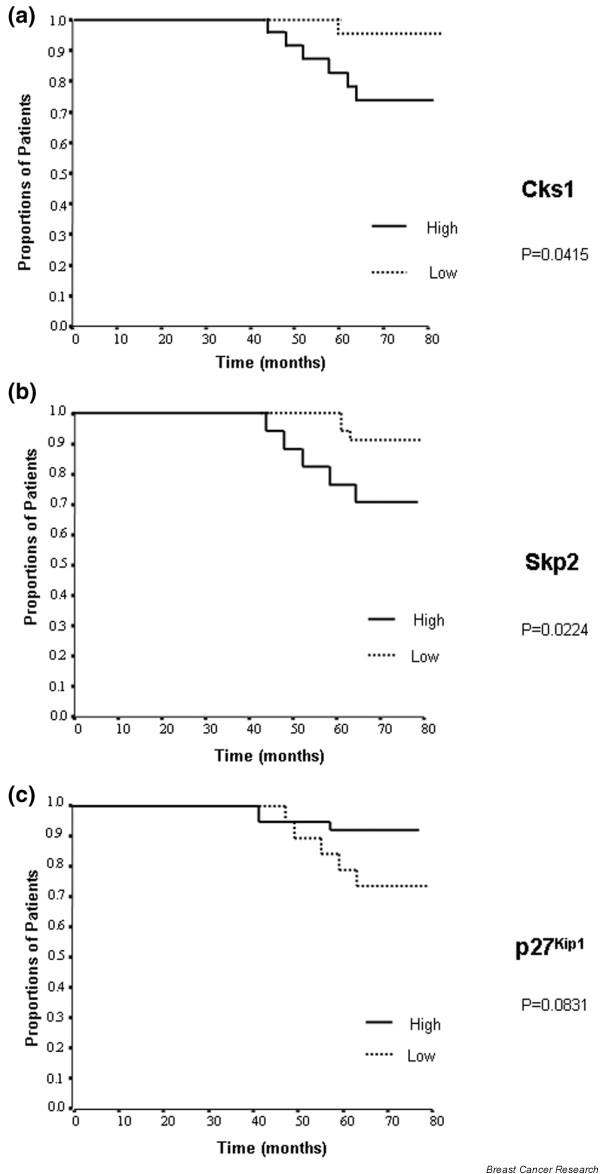
The association between expression of cell cycle proteins and survival of patients with breast cancer. Relation between Cks1 (cyclin kinase subunit 1)(a), Skp2 (S-phase kinase protein 2) (b), and p27^Kip1 ^(c) expression and overall survival (death). Analyses were constructed using the Kaplan–Meier method. Protein expression was considered low if fewer than 10% of cell nuclei were stained and high if more than 10% of nuclei were stained.

There was also a strong association between Cks1 expression and overall survival (Fig. 4a). The mean survival rate for patients with low Cks1 expression was 79 months, significantly higher than that (72 months) of patients with high Cks1 levels (*P = *0.0415). Similarly, Skp2 expression was also found to be a strong predictor of overall survival (69 months vs 79 months *P = *0.0224) (Fig. 4b), but p27^Kip1 ^expression was not (*P = *0.0831; Fig. 4c).

## Discussion

Recent studies have clearly shown that Cks1 plays an essential role in Skp2-mediated degradation of p27^Kip1^. The mechanistic model that emerges from these studies suggests that Cks1 is associated with Skp2 and confers an allosteric change in Skp2 that increases its affinity for phosphorylated p27^Kip1 ^[18]. As a result, the Cks1-Skp2 interaction enables efficient transfer of ubiquitin to p27^Kip1^, resulting in rapid proteasome-mediated degradation. Whereas the role of Skp2 as an oncogene responsible for down regulation of p27^Kip1 ^protein levels has been well established in a wide variety of cancers, the role of Cks1 in many of these cancers, and in particular breast cancer, is unknown. This study provides new insights into its role in human breast cancer. Using immunohistochemical studies obtained from tumor specimens of patients with nonmetastatic breast cancer, we found that Cks1 was overexpressed in subsets of patients with unfavorable histological features. Furthermore, its expression was strongly and independently associated with poor disease-free survival and poor overall survival. Similar findings were recently reported by our and other laboratories in aggressive gastric, lung, oral, and colorectal carcinomas [20-24]. Taken together, these results suggest that Cks1 has an important role in the decrease in p27^Kip1 ^levels in aggressive cancers.

The interaction between the three proteins was evident in a significant proportion of the study patients, whereby the expression of Cks1 was strongly associated with Skp2 expression and inversely with p27^Kip1^protein levels. However, in five patients high Cks1 levels were associated with low p27^Kip1 ^despite low Skp2 levels, and in three patients Cks1 levels were high but Skp2 and p27^Kip1 ^levels were low. These findings, in which the inverse relation between Cks1 and p27^Kip1 ^was stronger than that between Skp2 and p27^Kip1 ^levels, are particularly interesting, considering that Skp2 is the prime mediator of p27^Kip1 ^degradation. Although we do not yet know the reason for this observation, we postulate that in the presence of high Cks1 levels Skp2 degradation of p27^Kip1 ^is more efficient and that lower levels of Skp2 are therefore required to down-regulate p27^Kip1^.

Cks1 overexpression was also strongly associated with various clinical and pathological features that are commonly used to determine aggressive tumor behavior, including poor tumor differentiation and lack of receptors to estrogen and progesterone. The association between Cks1, Skp2, and p27^Kip1 ^and ER expression was also observed in breast carcinoma cell lines. Estrogen-dependent cell lines displayed lower levels of Cks1 and Skp2 than estrogen-independent cell lines. Nevertheless, estrogen modulation affected the levels of these proteins, suggesting that tamoxifen may have an important therapeutic role in ER-positive cancers expressing high levels of Cks1 or Skp2.

We found a strong correlation between young age of the patient and overexpression of either Cks1 or Skp2. Thus, these results support the notion that breast cancer has more aggressive tumor biology in young patients than in older ones, and that this difference may account, at least in part, for the poorer survival rates of younger patients than of older patients with similar clinicopathological features. Overall, Cks1 or Skp2 were each found to be good prognostic markers for disease-free and overall survival. Interestingly, there was no significant association between p27^Kip1 ^expression and overall survival (*P = *0.083). A possible reason for this finding is the relatively small number of patients in this study. Larger studies may be required to resolve this issue.

## Conclusion

The results of the present study provide additional insights into the importance of alterations in cell-cycle regulatory proteins and tumor progression in breast cancer. Our findings suggest that Cks1 has an important role in the deregulation of the cell-cycle protein p27^Kip1 ^in breast cancer. Overexpression of Cks1 was strongly associated with increased Skp2 expression and down regulation of p27^Kip1 ^levels, resulting in aggressive tumor behavior and poor prognosis. Thus, both Cks1 and Skp2 may be considered as potential novel prognostic markers and targets for the future development of specific therapeutic interventions in breast cancer.

## Abbreviations

Cks = cyclin kinase subunit; ER = estrogen receptor; PR = progesterone receptor; Skp = S-phase kinase protein; TBST = Tris-buffered saline and 0.1% Tween 20.

## Competing interests

The authors declare that they have no competing interests.

## Authors' contributions

MS (Merav Slotky) and MS (Ma'anit Shapira) contributed equally in the preparation of the study. Both carried out the immunohistochemical studies and the immunobot analysis, participated in the sequence alignment, and drafted the manuscript. OBI carried out the pathological and immunohistochemical analysis and interpretation. SL and BF participated in the design of the study and performed the statistical analysis. MT collected and analyzed the clinical data and helped draft the manuscript. DDH conceived of the study, participated in its design and coordination, and helped to draft the manuscript. All authors read and approved the final manuscript.

## Figures and Tables

**Table 1:** Relations between protein^a ^expression and clinicopathological characteristics in patients with breast cancer

**Characteristic**	**Cks1**			**Skp2**			**p27^Kip1^**		
	**High**	**Low**	***P* ^b^**	**High**	**Low**	***P* ^b^**	**High**	**Low**	***P* ^b^**

Grade									
1	2	8	0.012	2	8	0.005	2	8	0.032
2	14	14	0.012	7	21	0.005	14	14	0.032
3	10	2	0.012	9	3	0.005	8	4	0.032
Tumor size^c^									
T1	15	18	0.333	10	23	0.265	19	14	0.485
T2	10	6	0.333	7	9	0.265	8	8	0.485
T3	1	0	0.333	1	0	0.265	0	1	0.485
Lymph node involvement^c^									
N0	16	15	0.508	11	20	0.803	18	13	0.387
N1	6	3	0.508	4	5	0.803	3	6	0.387
N2	4	6	0.508	3	7	0.803	6	4	0.387
Estrogen receptor expression									
Negative	17	6	0.013	16	7	0.001	9	14	0.048
Positive	10	17	0.013	3	24	0.001	17	10	0.048
Progesterone receptor expression									
Negative	15	3	0.006	14	4	0.001	5	13	0.032
Positive	12	20	0.006	4	27	0.001	20	12	0.032
Age (years)									
>50	14	5	0.017	12	7	0.002	7	12	0.051
< 50	12	19	0.017	25	6	0.002	20	11	0.051

^a^Cks1, cyclin kinase subunit 1; p27^Kip1^, a cell-cycle inhibitory protein; Skp2, S-phase kinase protein 2. ^b^Refers to the differences between protein expression and the variables. ^c^According to the TNM (tumor, node, metastases) staging system of the American Joint Committee on Cancer. *P *≤ 0.05 (values shown in bold type) was considered statistically significant.

## References

[B1] Morabito A, Magnani E, Gion M, Sarmiento R, Capaccetti B, Longo R, Gattuso D, Gasparini G (2003). Prognostic and predictive indicators in operable breast cancer. Clin Breast Cancer.

[B2] Tsihlias J, Kapusta L, Slingerland J (1999). The prognostic significance of altered cyclin-dependent kinase inhibitors in human cancer. Annu Rev Med.

[B3] Alkarain A, Jordan R, Slingerland J (2004). p27 deregulation in breast cancer: prognostic significance and implications for therapy. J Mammary Gland Biol Neoplasia.

[B4] Chiarle R, Pagano M, Inghirami G (2001). The cyclin dependent kinase inhibitor p27 and its prognostic role in breast cancer. Breast Cancer Res.

[B5] Catzavelos C, Bhattacharya N, Ung YC, Wilson JA, Roncari L, Sandhu C, Shaw P, Yeger H, Morava-Protzner I, Kapusta L, Franssen E (1997). Decreased levels of the cell-cycle inhibitor p27Kip1 protein: prognostic implications in primary breast cancer. Nat Med.

[B6] Loda M, Cukor B, Tam SW, Lavin P, Fiorentino M, Draetta GF, Jessup JM, Pagano M (1997). Increased proteasome-dependent degradation of the cyclin-dependent kinase inhibitor p27 in aggressive colorectal carcinomas. Nat Med.

[B7] Tsihlias J, Kapusta LR, DeBoer G, Morava-Protzner I, Zbieranowski I, Bhattacharya N, Catzavelos GC, Klotz LH, Slingerland JM (1998). Loss of cyclin-dependent kinase inhibitor p27Kip1 is a novel prognostic factor in localized human prostate adenocarcinoma. Cancer Res.

[B8] Hommura F, Dosaka-Akita H, Mishina T, Nishi M, Kojima T, Hiroumi H, Ogura S, Shimizu M, Katoh H, Kawakami Y (2000). Prognostic significance of p27KIP1 protein and ki-67 growth fraction in non-small cell lung cancers. Clin Cancer Res.

[B9] Pagano M, Tam SW, Theodoras AM, Beer-Romero P, Del Sal G, Chau V, Yew PR, Draetta GF, Rolfe M (1995). Role of the ubiquitin-proteasome pathway in regulating abundance of the cyclin-dependent kinase inhibitor p27. Science.

[B10] Hershko A, Ciechanover A (1998). The ubiquitin system. Annu Rev Biochem.

[B11] Carrano AC, Eytan E, Hershko A, Pagano M (1999). SKP2 is required for ubiquitin-mediated degradation of the CDK inhibitor p27. Nat Cell Biol.

[B12] Sutterluty H, Chatelain E, Marti A, Wirbelauer C, Senften M, Muller U, Krek W (1999). p45SKP2 promotes p27Kip1 degradation and induces S phase in quiescent cells. Nat Cell Biol.

[B13] Tsvetkov LM, Yeh KH, Lee SJ, Sun H, Zhang H (1999). p27(Kip1) ubiquitination and degradation is regulated by the SCF(Skp2) complex through phosphorylated Thr187 in p27. Curr Biol.

[B14] Signoretti S, Di Marcotullio L, Richardson A, Ramaswamy S, Isaac B, Rue M, Monti F, Loda M, Pagano M (2002). Oncogenic role of the ubiquitin ligase subunit Skp2 in human breast cancer. J Clin Invest.

[B15] Hershko D, Bornstein G, Ben-Izhak O, Carrano A, Pagano M, Krausz MM, Hershko A (2001). Inverse relation between levels of p27(Kip1) and of its ubiquitin ligase subunit Skp2 in colorectal carcinomas. Cancer.

[B16] Gstaiger M, Jordan R, Lim M, Catzavelos C, Mestan J, Slingerland J, Krek W (2001). Skp2 is oncogenic and overexpressed in human cancers. Proc Natl Acad Sci USA.

[B17] Yang G, Ayala G, De Marzo A, Tian W, Frolov A, Wheeler TM, Thompson TC, Harper JW (2002). Elevated Skp2 protein expression in human prostate cancer: association with loss of the cyclin-dependent kinase inhibitor p27 and PTEN and with reduced recurrence-free survival. Clin Cancer Res.

[B18] Ganoth D, Bornstein G, Ko TK, Larsen B, Tyers M, Pagano M, Hershko A (2001). The cell-cycle regulatory protein Cks1 is required for SCF(Skp2)-mediated ubiquitinylation of p27. Nat Cell Biol.

[B19] Spruck C, Strohmaier H, Watson M, Smith AP, Ryan A, Krek TW, Reed SI (2001). A CDK-independent function of mammalian Cks1: targeting of SCF(Skp2) to the CDK inhibitor p27Kip1. Mol Cell.

[B20] Masuda TA, Inoue H, Nishida K, Sonoda H, Yoshikawa Y, Kakeji Y, Utsunomiya T, Mori M (2003). Cyclin-dependent kinase 1 gene expression is associated with poor prognosis in gastric carcinoma. Clin Cancer Res.

[B21] Inui N, Kitagawa K, Miwa S, Hattori T, Chida K, Nakamura H, Kitagawa M (2003). High expression of Cks1 in human non-small cell lung carcinomas. Biochem Biophys Res Commun.

[B22] Kitajima S, Kudo Y, Ogawa I, Bashir T, Kitagawa M, Miyauchi M, Pagano M, Takata T (2004). Role of Cks1 overexpression in oral squamous cell carcinomas: cooperation with Skp2 in promoting p27 degradation. Am J Pathol.

[B23] Shapira M, Ben-Izhak O, Bishara B, Futerman B, Minkov I, Krausz MM, Pagano M, Hershko DD (2004). Alterations in the expression of the cell cycle regulatory protein cyclin kinase subunit 1 in colorectal carcinoma. Cancer.

[B24] Shapira M, Ben-Izhak O, Linn S, Futerman B, Minkov I, Hershko DD (2005). The prognostic impact of the ubiquitin ligase subunits Skp2 and Cks1 in colorectal carcinoma. Cancer.

